# Exploiting artificial intelligence in precision oncology: an updated comprehensive review

**DOI:** 10.1186/s12967-025-07308-2

**Published:** 2025-12-16

**Authors:** Roaa Yousry Goda, Amal Kamal Abdel-Aziz

**Affiliations:** 1 PharmD Program, Faculty of Pharmacy, Ain Shams University, Cairo, 11566 Egypt; 2 Department of Pharmacology and Toxicology, Faculty of Pharmacy, Ain Shams University, Cairo, 11566 Egypt

**Keywords:** Artificial intelligence powered models, Anticancer drug discovery, Deep learning, Machine learning, Neural network, Precision cancer medicine

## Abstract

Precision oncology considers the genetic makeup of both the tumor and the cancer patient, medical history, clinical metadata, lifestyle and environmental factors. Thus, adoption of precision cancer medicine mandates sophisticated integrative analysis. Being a master in sorting and solving the puzzle pieces, artificial intelligence (AI)-powered frameworks emerged to fill the gap via executing multimodal analysis and generating meaningful outputs. Herein, we systematically discussed the opportunities and challenges of exploiting AI-assisted models in preclinical cancer research and in clinical oncology to promote precision medicine of cancer patients. We also shed light on the FDA-approved AI-driven tools and reviewed the clinical trials evaluating the capabilities of AI-supported algorithms in the diagnosis, screening, risk stratification, anticancer drug response prediction, clinical trial matching, informed treatment decision-making, personalized prescription and supportive care of cancer patients. Despite the foreseen promise of AI-driven frameworks in revolutionizing cancer personalized medicine, large-scale multicenter prospective studies and consensus regulatory guidelines are urged to ensure their safe, efficient and responsible use.

**Graphical Abstract:**

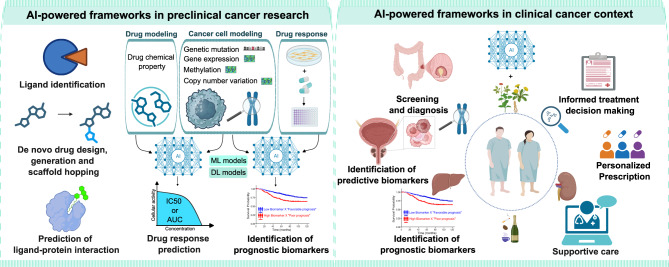

**Supplementary Information:**

The online version contains supplementary material available at 10.1186/s12967-025-07308-2.

## Key points

Precision cancer medicine mandates AI-powered integrative data analysis

AI-driven tools promotes anticancer drug discovery and development

AI-assisted models improve cancer screening, diagnosis and risk stratification

AI-supported frameworks guide informed treatment-decision making

Present limitations of AI-driven algorithms restrain their clinical utility

**Supplementary Information:**

The online version contains supplementary material available at 10.1186/s12967-025-07308-2.

## Cancer precision and personalized medicine

Cancer precision medicine tailors treatment based on the patient’s tumor, the patient’s genetic makeup, medical record, patient’s characteristics, lifestyle and environmental factors [1–3]. Precision oncology aims at optimizing the clinical outcome of anticancer therapy, preventing the adverse effects, and making healthcare more predictive, personalized, and cost-effective [3–5]. The pros and cons of precision medicine are extensively reviewed elsewhere [3–7]. In this review, we highlight the gap between the expected and real-life scenarios of exploiting targeted therapies and pharmacogenomics in cancer therapy. Artificial intelligence (AI) mimics human intelligence and has broad applications in various fields [8–13]. Machine learning (ML), a subset of AI, trains machines to automatically learn from the data by recognizing patterns and then make predictions based on the learnt patterns [11, 14]. There are several ML algorithms exploited in medicine including linear regression algorithms, logistic regression algorithms and decision trees and random forests algorithms [14, 15]. Artificial neural network (ANN), a ML algorithm, resembles the architecture and function of the biological neural networks of the brain. ANNs comprise nodes (i.e. simulating the neuronal cell bodies) linked via weighted connections (i.e. mimicking the axons and dendrites) [14]. The nodes of ANN receive inputs (data such as text or images), identify patterns and promote decision making [2, 14]. ANNs have a single hidden layer which connects the input layer with the output layer. In contrast, deep neural networks (DNNs), another subset of ML, consist of multiple hidden layers (i.e. deep) which significantly enhance their accuracy and capability in terms of detecting features [14, 16]. AI is deemed to transform the precision medicine of cancer patients [8, 17]. Herein, we critically discuss the potential and limitations of AI-driven tools within: i) preclinical cancer research as well as ii) clinical oncology settings to fulfill the promise of cancer precision and personalized therapy.

## Targeted therapy: Does it fulfill the promise of cancer precision medicine?

Exploiting the state-of-the-art technologies deepened our comprehension of the molecular mechanisms underlying the genetic and non-genetic aberrations implicated in tumorigenesis and cancer resistance [18–24]. Indeed, the treatment paradigm of diverse cancer types shifted from conventional chemotherapy to targeted therapy, a form of precision medicine [25–27]. In 2001, imatinib, the first-in-class tyrosine kinase inhibitor (TKI), was approved by the Food and Drug Administration (FDA) for treatment of mutated BCR-ABL (breakpoint cluster region (BCR) and abelson murine leukemia viral oncogene homolog 1 (ABL1)) chronic myeloid leukemia (CML) [28, 29]. Although imatinib revolutionized the treatment of BCR-ABL CML patients, resistance to imatinib has been reported [28, 30–32].

Several potent small molecules were developed to selectively target tyrosine kinase receptors such as epidermal growth factor receptor (EGFR), human epidermal growth factor receptor 2 (HER2) and FMS-like tyrosine kinase 3 (FLT3) which are overexpressed or constitutively activated in distinct types of cancer including breast cancer, lung cancer, bladder cancer, stomach cancer and acute myeloid leukemia (AML) [24, 33–36]. Nonetheless, cancer resistance to TKIs as well as TKI-associated toxicities largely restrained their clinical efficacy [21, 37–42].

Antibody-based therapy is another type of targeted cancer therapy [40, 43–49]. Bevacizumab is an anti-vascular endothelial growth factor (anti-VEGF) developed to suppress angiogenesis [40, 50]. Immune checkpoint inhibitors (ICIs) are monoclonal antibodies directed against the inhibitory brakes of the immune system including the cytotoxic T-lymphocyte-associated protein 4 (CTLA4), programmed cell death protein 1 (PD1) and programmed cell death ligand 1 (PDL1) [44, 51–53]. Notably, only a modest proportion of the ICI-treated cancer patients undergo complete remission and the vast majority of the treated cancer patients suffer from the autoimmune related adverse effects of ICIs which are dose-limiting and mandate treatment interruptions [44, 52, 54, 55].

Antibody-drug conjugates (ADCs) permit preferential delivery of cytotoxic agents to tumor cells [49, 56]. Trastuzumab emtansine and trastuzumab deruxtecan are HER2-targeted ADCs which elicited clinical benefit in patients with HER2^low^ and HER^high^ breast cancer [49, 56, 57]. Nonetheless, resistance to HER2-targeted ADCs was reported. Trastuzumab deruxtecan and to a lower extent trastuzumab emtansine were associated with drug-related interstitial lung disease or pneumonitis [58].

Thus, given their selectivity and relatively fewer adverse effects compared to conventional chemotherapy, targeted therapies were foreseen to render cancer “a curable disease”. Nonetheless, intrinsic and acquired cancer resistance as well as the specific dose-limiting toxicities of targeted therapies restrained their anticancer capability.

## Pharmacogenomics: Does it fulfill the promise of cancer precision and personalized medicine?

Pharmacogenomics emerged as a fundamental field which has the potential to unleash – at least partially – the therapeutic potential of precision medicine [59–61]. Pharmacogenomics investigates how the genetic makeup modulates patient’s response to therapy in terms of pharmacokinetics (e.g. metabolism), pharmacodynamics, and toxicokinetics/toxicodynamics [59, 62].

Methemoglobinemia and hemolytic anemia were reported in cancer patients with glucose-6-phosphate dehydrogenase (G6PD) deficiency following their treatment with rasburicase [59, 63, 64]. The latter is contraindicated in G6PD deficient patients [59, 63, 64]. Rasburicase – an oxidizing drug – is a uric acid oxidase (uricase) which converts uric acid to the more soluble metabolite “allantoin” and is used in the treatment of tumor lysis syndrome [59, 63, 64]. The Clinical Pharmacogenetics Implementation Consortium (CPIC) has published recommendations for adjusting the doses of three thiopurines (azathioprine, mercaptopurine, and thioguanine) based on thiopurine methyl transferase (TPMT) and nudix hydrolase 15 (NUDT15) genotypes [65]. Variant alleles of TPMT and NUDT15 with low enzymatic activities are associated with higher levels of thiopurines and/or their cytotoxic metabolites and hence myelosuppression [59, 65].

Cancer pharmacogenomics evolved from the traditional perception of pharmacogenetics linked to specific drug-gene pairings, to include the state-of-the-art next generation sequencing (NGS) technologies such as whole exome sequencing (WES) or whole genome sequencing (WGS) which offer a more holistic “non-biased” analysis and hence could identify novel common and rare variants [1]. Nonetheless, WES and WGS are still challenged by their relatively high costs and problems linked to the storage, processing and interpretation of large amounts of data generated [66, 67].

## Exploiting artificial intelligence (AI)-driven tools in precision oncology

Given its multimodal architecture, AI is expected to transform precision medicine [2, 8, 68–74]. AI-driven algorithms have been integrated into NGS technologies including genomics, epigenomics, variant calling, transcriptomics, proteomics and single-cell sequencing [9]. AI-powered tools such as ML and DL optimized each step of the NGS workflow starting from the experimental design, library preparation as well as bioinformatics analysis [9, 75]. AI-driven tools also processed and analyzed large datasets including that of electronic health records, diagnostic imaging and biomarkers [2, 8, 68, 76]. AI-assisted gene editing tools as Clustered Regularly Interspaced Short Palindromic Repeats (CRISPR)-Cas9 promoted efficient targeted genome modifications [75]. The AI-driven AlphaFold neural network model integrated physical and biological knowledge about protein structure to accurately predict the three-dimensional (3D) structure of proteins with no similar known structures [77]. The technical algorithms and workflows of AI-based tools are extensively reviewed elsewhere [2, 9]. Herein, we discussed the lessons learnt from exploiting AI-driven tools in both preclinical cancer research as well as clinical oncology.

### Preclinical applications of AI-driven tools in basic and translational cancer research

Cancer driver genes are the genes whose mutations confer proliferative advantage to the affected cell [78]. Comprehensive PanCancer analysis using AI-driven algorithms identified 299 driver genes and more than 3000 putative mis-sense driver mutations across diverse types of cancer [78]. Identification of actionable driver mutations - for which targeted therapies exist - is of paramount importance for guiding clinicians towards precision medicine.

PandaOmics is a cloud-based platform which exploits AI and bioinformatic tools to analyze multi-omics datasets as well as biomedical text datasets to ultimately promote the discovery of biomarkers and therapeutic targets [79, 80]. It also comprises an inClinico platform which predicts the probability of the transition of a Phase 2 clinical trial to Phase 3 [79]. Via exploiting PandaOmics, Chueng and colleagues pinpointed that voltage-dependent calcium channel subunit alpha-2/delta-1 (CACNA2D1) might act as a potential therapeutic target in Epstein-Barr virus positive (EBV^+^) nasopharyngeal cancer [80]. Tethering of the EBV episome (or genome) increased H3K27 acetylation – which is a marker for active enhancers and promoters – in proximity to the promoter of CACNA2D1 gene in EBV^+^ nasopharyngeal cancer cells. Indeed, nasopharyngeal cancer expressed higher CACNA2D1 transcript and protein levels compared to normal nasopharyngeal samples. Genetic depletion of CACNA2D1 in EBV^+^ nasopharyngeal cancer cell line evidently reduced its proliferation in vitro and in vivo [80].

Within the context of structure-based drug discovery, AI-powered tools were used for ligand identification, *de novo* drug design and prediction of the ligand-protein binding pose and potency [75, 81]. MolProphet is a cloud-based AI-driven drug discovery platform which promoted target pocket prediction, structure-based and ligand-based virtual screening, hit discovery, lead optimization, *de novo* molecule generation and scaffold hopping [82]. Pandey utilized MolProphet platform to generate novel small molecules targeting aurora kinase A (AURKA) which is overexpressed in diverse types of cancer and acts as a synthetic lethal partner of several tumor suppressor genes [83–85]. Molecular simulations, molecular dynamics analysis, and binding energy calculations suggested that – out of 200 novel compounds − 4-(5-fluoro-6-[(1Z)-3-hydrazinyl-3-oxo-2-phenylprop-1-en-1-yl]pyridin-2-yl)benzoic acid could be a candidate AURKA inhibitor. However, this study relied only on in-silico simulation and in vitro validation is warranted [83]. AI-driven tools also have the potential to advance the field of polypharmacology (which refers to small molecules interacting with multiple targets simultaneously) through predicting their selectivity and off-target effects. Nonetheless, experimental validation methods are needed to confirm the selectivity and absence of off-target effects [86].

Artificial intelligence is also speculated to optimize the therapeutic outcome of antibody-based cancer therapy [2, 87]. Within the context of chimeric antigen receptor (CAR) T-cell therapy, AI-assisted platforms are foreseen to optimize target identification, vector design, manufacturing and personalized data-driven clinical decisions [87].

**Fig. 1:**
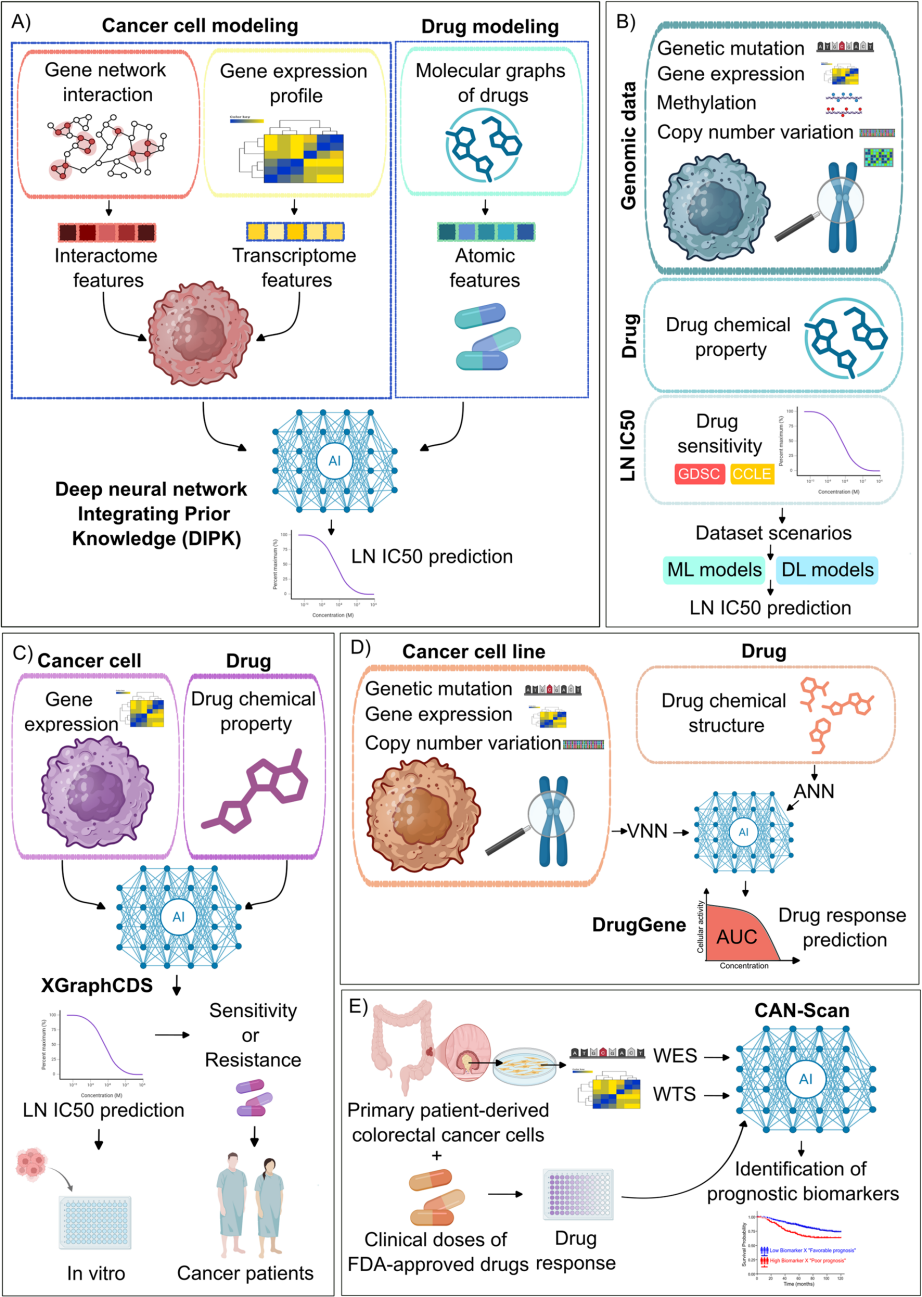
Simplified illustration of the AI-powered frameworks exploited in preclinical cancer research. A) Deep neural network Integrating Prior Knowledge (DIPK) framework was constructed to predict the drug responsiveness of cancer cell lines [88]. DPIK integrates: i) cell modeling: the interactome features were captured from the gene interaction network and the transcriptome features were obtained from gene expression profiles of cancer cell lines and ii) drug modeling: the atomic features were derived from the atomic and chemical bonding details of a drug. The output of DIPK was the prediction of the logarithm of the median inhibitory concentration (LN IC50).B) Schematic representation of the machine learning (ML) and deep learning (DL) models which integrated multi-omics datasets (including genetic mutation, gene expression, DNA methylation and copy number variation (CNV), drug chemical properties and drug sensitivity (LN IC50) of cancer cell lines treated with drugs (obtained from Genomics of Drug Sensitivity in Cancer (GDSC) and Cancer Cell Line Encyclopedia (CCLE)) as learning data for predicting anticancer drug responsiveness (IC50) [90]. C) “XGraphCDS” comparative learning framework was developed to predict differential cancer responses to pharmacotherapy. This model was based on cancer gene expression profile and the chemical structure knowledge of the drugs. The output was quantitative (LN IC50) or qualitative (sensitivity or resistance). An in vivo prediction model for cancer patients was constructed from the in vitro data [91]. D) Workflow of “DrugGene” framework. This model combined two types of neural networks: i) visual neural network (VNN) which integrated the mutational landscape, CNV and gene expression profiles of cancer cells and ii) artificial neural network (ANN) which input the chemical structural features of anticancer drugs. The output of DrugGene was the predicted area under the curve (AUC) where 0 and 1 reflected complete cell killing and no effect respectively [92]. E) Workflow of the CAN-Scan platform. Primary patient-derived colorectal cancer cells were characterized for their drug response using clinically relevant doses of FDA-approved drugs, whole exome sequencing (WES) and whole transcriptome sequencing (WTS). ML algorithms were used to train drug-specific predictive models to identify prognostic biomarkers. This figure was prepared at least partially using Biorender software

Deep neural network Integrating Prior Knowledge (DIPK) - a DL-based framework - was developed to predict the susceptibility of cancer cell lines to pharmacological agents (Fig. 1A). DIPK comprised: i) cell modeling: which was acquired from the gene interaction (or interactome features) as well as gene expression (or transcriptome features), ii) drug modeling: the molecular graphs of drugs were obtained from the atomic and chemical bonding details of the drugs extracted from their Simplified Molecular Input Line Entry System (SMILES) string, iii) fusion of the interactome features, transcriptome features and atomic features and iv) output which was the prediction of the logarithm of the median inhibitory concentration (LN IC50) [88]. The performance of DIPK was evaluated using the Genomics of Drug Sensitivity in Cancer (GDSC) dataset. GDSC is a comprehensive resource that includes the gene expression profiling of cancer cell lines together with their IC50 to distinct drugs [92]. Notably, there was a strong correlation between the predicted and observed LN IC50 values [88]. DIPK also predicted drug response at a single-cell level. Moreover, DIPK forecasted better response of breast cancer patients to paclitaxel within the pathologic complete response cohort compared to the residual disease cohort. Nevertheless, further systematic large-scale prospective studies are needed to evaluate the potential of incorporating DIPK in decision-making and devising personalized treatment strategies for the people of cancer [88].

To de-noise and mitigate the inconsistencies observed with drug sensitivity data matrices including that of Cancer Therapeutics Response Portal (CTRP) and GDSC, Hu and colleagues introduced a DL framework called residual threshold deep matrix factorization (RT-DMF) [93]. RT-DMF yielded corrected datasets which better correlated with each other and conferred better predictive potential than the original datasets [93].

Regression-based ML and DL models were constructed to predict anticancer drug response (LN IC50) by using the IC50 of cancer cell lines treated with drugs (from the GDSC and the Cancer Cell Line Encyclopedia (CCLE)), multi-omics datasets (including that of genetic mutation, gene expression, DNA methylation and copy number variation (CNV)) and drug chemical properties (Fig. 1B). The performance of DL models outperformed that of ML [89]. DL frameworks comprised two convolutional neural network (CNN) models: i) Cancer Drug Response profile scan (CDRScan) and ii) residual neural network (ResNet). CDRScan was based on a drug screening assay supported by the genomic somatic mutational landscape of 787 human cancer cell lines and structural profiles of 244 drugs [94]. DNNs are challenged with the vanishing gradient problem which takes place when gradients computed during backpropagation become very small as they move backward across layers. This hinders the learning capability of the early layers which are closer to the input and thus, restrains effective training of the network. Notably, ResNet introduced the so-called “residual connections” which ameliorated the gradient vanishing problem, captured more complex features and permitted more efficient training of deeper networks. Indeed, the predictive capability of anticancer drug responsiveness using ResNet was better or comparable to that of CDRScan [89].

DeepDR, a DNN model, was developed to predict tumor response to drug therapy [95]. The model was trained using mutational and transcriptomics data of human cancer cell lines deposited in the GDSC database and ultimately applied to The Cancer Genome Atlas (TCGA) data to predict a patient tumor’s response to anticancer drugs [92, 95]. Indeed, DeepDR forecasted the IC50 values of 265 drugs and the drug responses of 33 cancer types (e.g. EGFR inhibitors in non-small lung cancer and. vinorelbine for titin-mutated tumors) [95].

Wang and colleagues formulated another neural network known as “XGraphCDS” to predict differential cancer responses to pharmacotherapy [90]. This comparative learning framework was based on cancer gene expression profile (from the GDSC and CCLE) and the chemical structure knowledge of the drugs. The output was quantitative (LN IC50) or qualitative (sensitivity or resistance) (Fig. 1C). An in vivo prediction model for cancer patients was constructed from the in vitro data [90].

Although IC50 is widely used as a prediction variable in regression models, more concrete measures such as the area under the curve (AUC) and area above the dose response curve (AAC) were proposed to be more robust [96]. Relying on continuous values rather than discrete (single) values of the IC50 was also recommended [96]. “DrugGene” - a DL model - was generated to predict the drug sensitivity via combining two types of neural networks: i) visual neural network (VNN) which modelled the hierarchical molecular subsystems within cancer cells via integrating their mutational landscape, CNV and gene expression profiles and ii) artificial neural network (ANN) which input the chemical structural features of anticancer drugs (Fig. 1D). The output of the predicted anticancer drug sensitivity was in the form of normalized AUC where 0 and 1 reflected complete cell killing and no effect respectively [91].

Kuenzi and colleagues developed a DL model called “DrugCell” which comprised two neural networks [97]. The first neural network simulated the intracellular hierarchical organization and identified which cellular subsystems (e.g. pathways and organelles) contributed to the anticancer responses (expressed as AUC) taking into consideration the mutational profile of cancer cells [97]. The second neural network integrated the chemical structure of the drugs [97]. It is worth mentioning that “DrugCell” predicted potentially effective comboregimens for AML therapy as lenalidomide and DNA methyl transferase (DNMT) inhibitors. Although meta-analysis suggested that lenalidomide + azacitidine (DNMT inhibitor) might be superior to azacitidine monotherapy in AML therapy, randomized clinical trial (RCTs) negated the additional beneficial effect of this combination [98, 99]. Thus, identifying predictive biomarkers are needed to identify the subset of the AML patients who are most likely to benefit from this comboregimen.

Many preclinical cancer therapies fail during clinical trials [100]. To better mimic clinical scenarios, Chia and colleagues presented CAN-Scan, which exploited ML to integrate the drug response signatures of primary patient-derived colorectal cancer cells tested against clinically relevant doses of 84 FDA-approved drugs with their multi-omics characterization (e.g. genetic alterations and transcriptomic signatures) [101]. Importantly, CAN-Scan linked overexpression of cell adhesion gene, increased BRAF/mitogen-activated protein kinase (MAPK) signaling as well as focal copy-number gain/amplification in chromosome 7 to the resistance of colorectal cancer to 5-fluorouracil [101]. Although CAN-Scan did not consider the potential impact of the tumor microenvironment (e.g. immune cells and fibroblasts) on cancer response, it accurately predicted the response of four cohorts of ethnically distinct colorectal cancer patients [101]. Drugs such as regorafenib and vemurafenib were proposed as a potential therapeutic option for treating BRAF^+^ CRC patients who are irresponsive to 5-fluorouracil [101]. Nonetheless, large-size multicenter prospective studies are warranted to validate this postulation.

### Clinical applications of AI-powered frameworks in cancer management

**Fig. 2:**
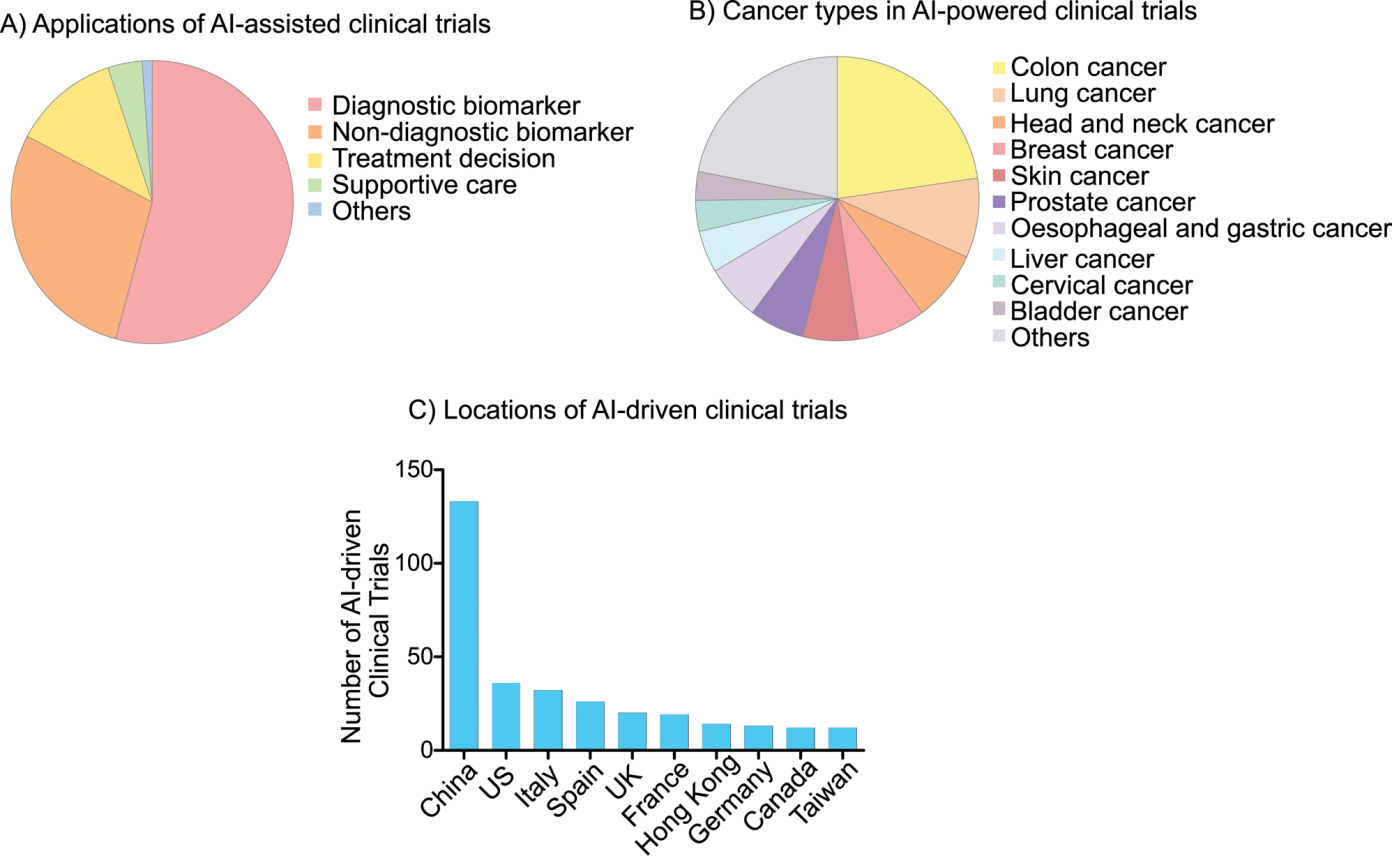
Applications, cancer types and locations of artificial intelligence (AI)-assisted clinical trials registered on ClinicalTrials.Gov database. (**A**) Applications of AI-powered tools in clinical trials (*n* = 397): diagnostic biomarker (~54%), non-diagnostic biomarker (28.6%), treatment decisions (~12%), supportive care (~4%) and other applications. (**B**) Cancer types in AI-assisted clinical trials [colon cancer (~22.7%), lung cancer (9%), head and neck cancer (8%), breast cancer (7.8%), skin cancer (6.3%), prostate cancer (6.3%), oesophageal cancer and gastric cancer (6.3%), liver cancer (~5%), cervical cancer (~3.5%) and bladder cancer (~3.3%)]. (**C**) Top ten countries with the largest number of clinical trials registered on ClinicalTrials.Gov database which evaluated the utility of AI-driven tools in cancer management

To obtain a broader overview of the applications of AI-driven tools in clinical oncology, we systematically searched the registered ClinicalTrials.gov studies which exploited AI-assisted tools (Supplementary Table 1). After omitting the false-positive search trials, 397 clinical trials were spotted; more than 50% of which evaluated the potential utility of AI-assisted frameworks in cancer diagnosis (Fig. 2A). Approximately 28.6 and 12% of the 397 clinical trials aimed to assess the potential utility of AI-powered tools for non-diagnostic purposes and for guiding treatment decisions respectively (Fig. 2B). The vast majority of the AI-assisted clinical trials were carried out in China (~33.5%) followed by the US (~9%) and Italy (~8%) (Fig. 2C). In Table 1, we shed light on selected AI-driven tools approved by the FDA to be used for cancer patients [17]. This section reviewed the real-world data of AI-powered models deployed in the screening, diagnosis, prediction of drug response, clinical trial matching, guiding informed treatment decisions as well as supportive care of cancer patients.

**Table 1:** Selected list of artificial intelligence (AI)-enabled tools approved by the Food and Drug Administration (FDA) for marketing in the United States

Device	Description
Computer-assisted diagnostic software for lesions suspicious for cancer	It characterizes lesions based on the features extracted from the images of magnetic resonance, mammography, radiography, or computed tomography.
Radiation Planning Assistant (RPA)	An AI-assisted tool that provides automated contouring and planning solutions for the treatment of several tumor and non-tumor specific radiotherapy indications. It is specifically designed for use in radiotherapy providers in low-income and middle-income countries.
SpotLight/SpotLight Duo with Low Dose Lung Cancer Screening Option	The low dose computed tomography (CT) X-ray screening option uses low dose CT for the screening and diagnosis of lung cancer.
Pathwork® Tissue of Origin Test Kit-FFPE (Formalin-Fixed Paraffin-Embedded)	An in vitro diagnostic test which assesses the degree of similarity between the gene expression patterns in a patient’s FFPE tumor and the gene expression profiles in a database of fifteen tumor types (including bladder cancer, breast cancer, colorectal cancer, gastric cancer, testicular germ cell cancer, hepatocellular cancer, kidney cancer, non-small cell lung cancer, non-Hodgkin’s lymphoma, melanoma, ovarian cancer, pancreatic cancer, prostate cancer, sarcoma, and thyroid cancer).
Avenda Health AI Prostate Cancer Planning Software “Unfold AI”	An AI-based software which integrates patient data and deep learning algorithms to enhance the accuracy of the diagnosis and treatment planning of prostate cancer.
Opulus™ Lymphoma Precision	An AI-powered software which analyzes whole-body fluorodeoxyglucose positron emission tomography/computed tomography (FDG-PET/CT) scans of patients with FDG-avid lymphomas (i.e. with high levels of absorbed FDG and increased metabolic activity).
TumorSight Viz	An AI-driven 3D visualization and analysis tool of breast MRI images from early stage or locally advanced breast cancer patients.
ArteraAI Prostate test	An AI-driven risk stratification tool for patients with non-metastatic prostate cancer.
QUIBIM Precision Prostate (qp-Prostate)	An AI-assisted tool used to enhance the detection and diagnosis of prostate cancer.
GI Genius™ system	An AI-assisted colonoscopy tool designed to help endoscopists detect colorectal polyps and colon adenomas.

AI: artificial intelligence, CT: computed tomography, FDG-PET: fluorodeoxyglucose positron emission tomography, FFPE: formalin-fixed paraffin-embedded, RPA: radiation planning assistant

#### Exploiting AI-driven tools as biomarkers in cancer patients

There are distinct types of biomarkers including diagnostic, monitoring, pharmacodynamic/response, predictive, prognostic, safety, and susceptibility/risk biomarkers [102–104]. Most of the Clinicaltrials.gov studies evaluated the potential added value of co-implementing AI-powered tools in cancer screening and diagnosis with a peculiar interest in colorectal cancer (Supplemental Table 1). Herein, we highlighted the lessons learnt from exploiting AI-supported frameworks as biomarkers.

**Fig. 3:**
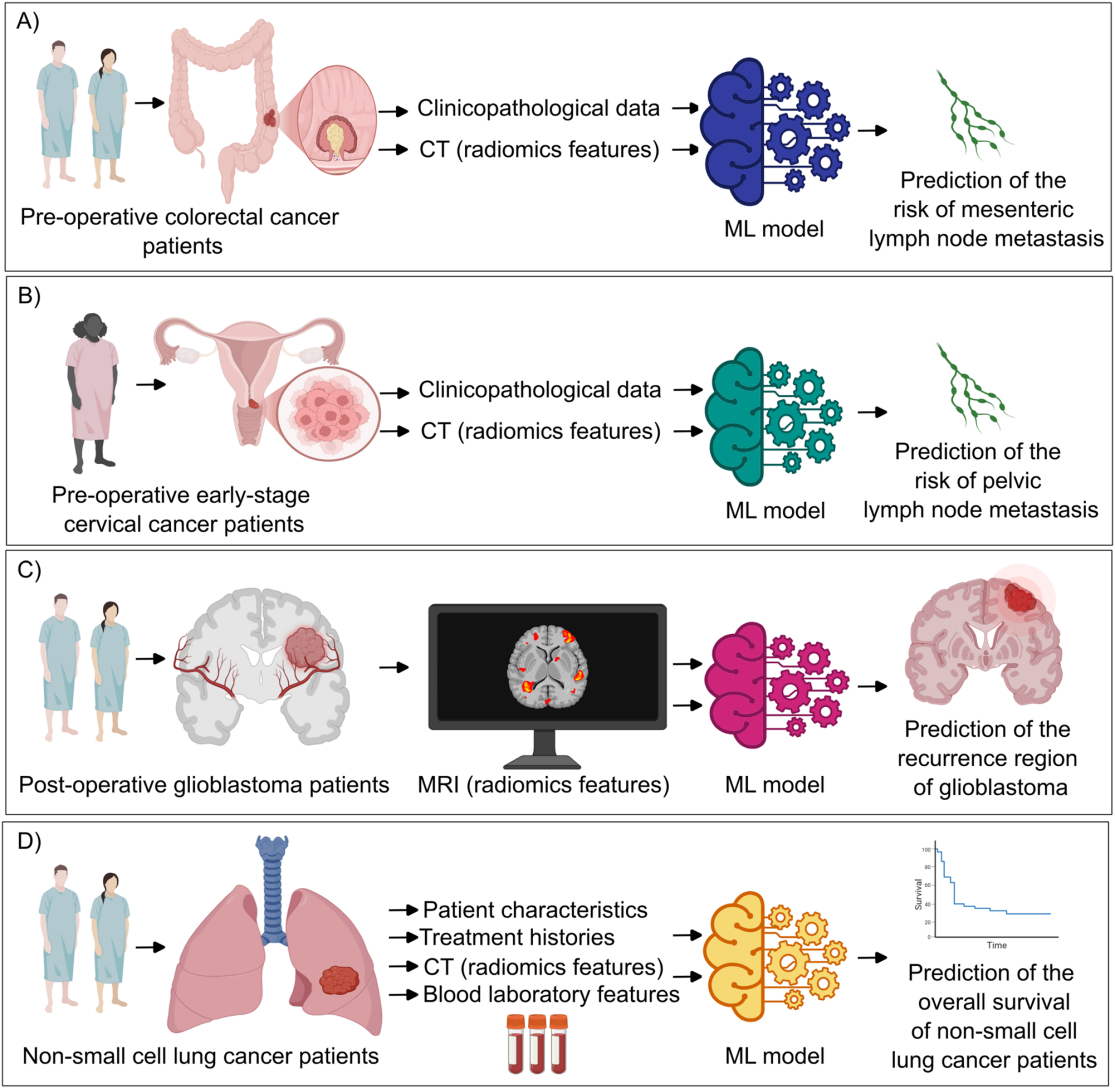
Schematic diagram of distinct applications of artificial intelligence (AI)-powered models which integrated radiomics features. (**A**) Machine learning (ML) models were based on integrated computed tomography (CT) image measurements and clinicopathological data (including blood tests and primary tumor pathology) from pre-operative colorectal cancer patients to predict the risk of mesenteric lymph node metastasis [107]. (**B**) ML models were developed to predict pelvic lymph node metastasis in pre-operative early-stage cervical cancer patients via integrating the pre-operative CT images of the whole abdomen and pelvis and clinicopathological data [108]. (**C**) ML models were developed to predict the location of recurrences of glioblastoma from post-operative MRI scans of glioblastoma patients [109]. (**D**) ML-assisted framework integrated basic non-small lung cancer, patient characteristics, histology, PDL1 tumor proportion score, driver oncogene status, patient’s anticancer treatment histories, blood laboratories findings and radiomics features of the primary tumor from contrast-enhanced CT images to predict personalized overall survival [110]. This figure was prepared at least partially using Biorender software

Radiomics refers to the extraction of quantitative phenotypic features from radiological images in an automated, high-throughput manner [105]. These features are then incorporated into AI-driven tools or statistical tools to perform a classification task to promote risk stratification, prognosis prediction or response prediction [105]. A ML model was constructed based on the clinical data and “radiomics signature” stemming from the imaging features from pelvic magnetic resonance imaging (MRI) to predict inguinal lymph node metastasis in pre-operative patients with vulvar cancer [106]. ML models integrated computed tomography (CT) image measurements and clinicopathological data (including blood tests and primary tumor pathology) from pre-operative colorectal cancer patients to ultimately predict the risk of mesenteric lymph node metastasis (Fig. 3A) [107]. Similarly, ML models were developed to predict pelvic lymph node metastasis in pre-operative early-stage cervical cancer patients via combining the pre-operative CT measurements of the whole abdomen and pelvis and clinicopathological data (Fig. 3B) [108]. External validation of these models is warranted to ensure their generalizability.

Meta-analysis of 9 studies exploiting AI-driven models in the diagnosis of soft tissue sarcoma revealed their superior accuracy and sensitivity [111]. Since the analyzed studies were retrospective, large size multicenter prospective studies are needed to test the generalizability of the results.

Kang and colleagues developed a DL model which accurately measured the volume of meningioma based on contrast-enhanced MRI scans [112]. Discriminating edema from glioblastoma is challenging [109]. To this end, a multi-institutional retrospective study by Cepeda and colleagues developed a ML model which distinguished glioblastoma from edema and predicted the location of tumor recurrences from post-operative MRI scans of glioblastoma patients (Fig. 3C). However, this study lacked anatomopathologic confirmation of the so-labeled recurrences areas and hence cannot exclude pseudoprogression [109].

Radiomics models supported by ML algorithms were also constructed to predict disease-free survival and overall survival in patients with locally advanced cervical cancer after concurrent chemoradiotherapy [113]. Despite an increasing number of studies evaluating the use of AI-driven algorithms to promote the response predictive capability of radiomics, these tools are still not widely adopted in clinical practice. This could be – at least partially - due to the lack of large-scale multicenter studies and standardized methods that do not enable a meaningful meta-analysis [114].

Koyama and colleagues developed an AI-based personalized survival prediction model according to treatment selection for patients with advanced non-small cell lung cancer (NSCLC) (Fig. 3D). This ML-assisted framework integrated baseline patient characteristics including age, sex, stage, histology, PDL1 tumor proportion score, driver oncogene status (as EGFR mutation, ALK fusion, ROS proto-oncogene 1 receptor tyrosine kinase (ROS1) fusion, and B-Raf proto-oncogene serine/threonine kinase (BRAF) mutation), patient’s anticancer treatment histories, laboratories findings and radiomics features of the primary tumor from contrast-enhanced CT images [110].

Probe based confocal laser endomicroscopy promotes accurate diagnosis of esophageal squamous neoplasms [115]. Nonetheless, analysis of probe based confocal laser endomicroscopy images mandates extensive experience. An AI-aided confocal laser endomicroscopy system displayed higher sensitivity compared to expert endoscopists and boosted the accuracy and sensitivity of non-expert endoscopists in the diagnosis of esophageal squamous neoplasms [115]. A RCT (trial registration ChiCTR2100044126) demonstrated that AI-assisted endoscopy (using DL algorithms) significantly boosted the detection rate of esophageal cancer and precancerous lesions compared to unassisted endoscopy and was safe [116].

Recognition of the intra-abdominal metastasis in advanced gastric cancer by the AI-aided laparoscopic exploration system matched that by novice (inexperienced) surgeons, but outperformed novice surgeons in identifying small lesions [117]. An international multicenter study revealed that real-time polyp assessment during colonoscopy using AI-aided diagnosis did not significantly increase the sensitivity for small neoplastic polyps compared with visual inspection [118].

Changes in the tongue features may reflect GIT diseases especially in the stomach [115]. Tian and colleagues reported that integrating ML with tongue diagnosis analysis might serve as an non-invasive tool for early detection of gastric cancer specifically in resource-limited healthcare facilities [115]. ML models were trained using baseline clinical data, tongue features (e.g. color, coating and texture), and tumor marker levels. Nonetheless, external validation and technical standardization of this AI-assisted tool are warranted.

**Fig. 4:**
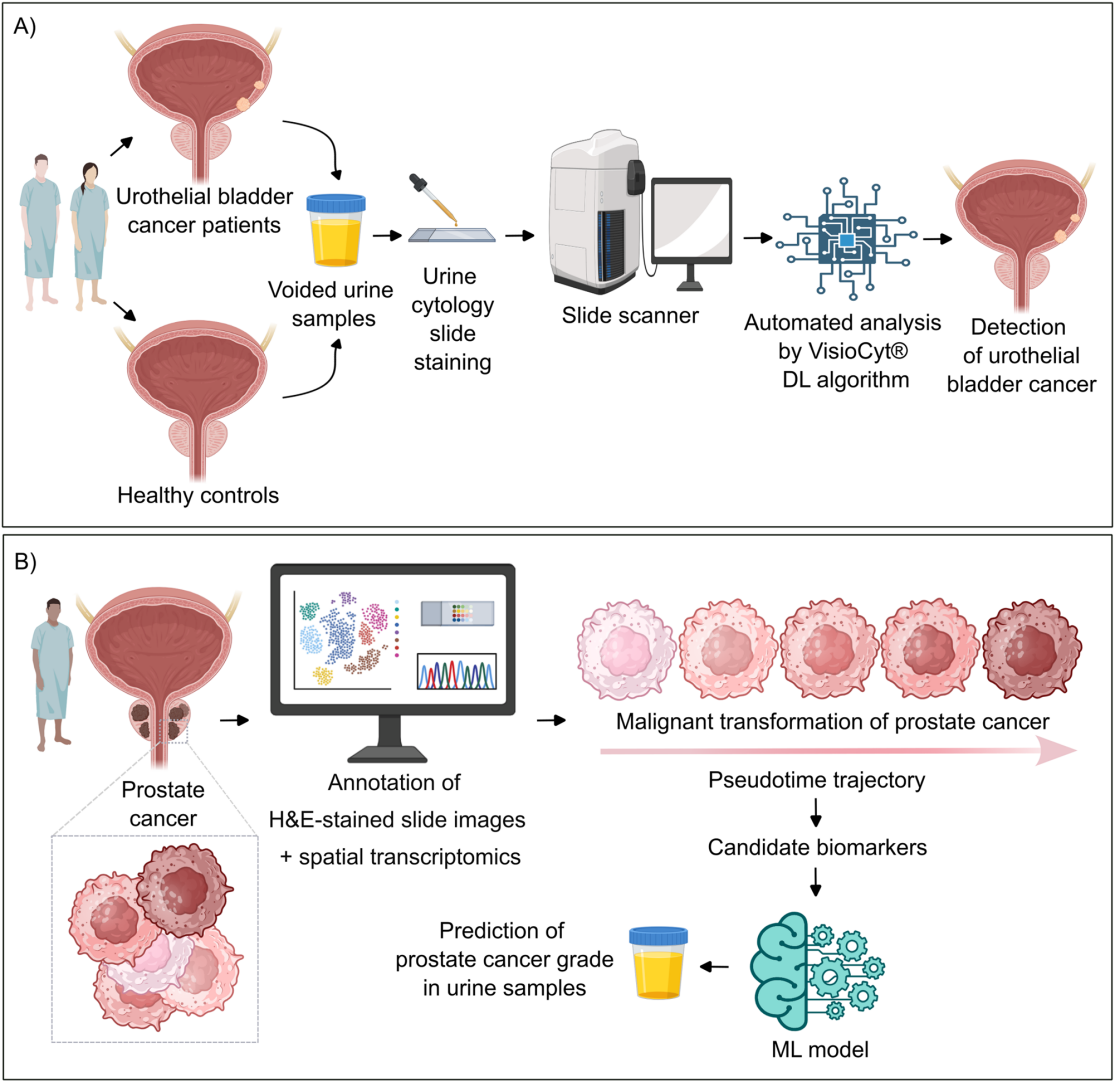
Simplified illustration of the workflow of artificial intelligence (AI)-driven frameworks used for the detection of cancer and its grade. (**A**) Simplified illustration of the workflow of VisioCyt®, an AI-assisted medical device. This deep learning (DL) algorithm automatically analysed digital cytology slides of voided urine samples to ultimately promote the detection of urothelial bladder cancer [119, 120]. (**B**) Spatial transcriptomics, pseudotime trajectory and machine learning (ML) algorithms were exploited to identify biomarkers which predicted the grade of prostate cancer. This figure was prepared at least partially using Biorender software

To diagnose bladder cancer, urologists rely on two fundamental tests: cystoscopy and cytology [119]. Many patients abandon cystoscopy given its invasive nature. Although voided cytology – which involves cell morphological analysis of urine biospecimens - is simple, non-invasive and thereby widely accepted by the patients, it mandates highly experienced uropathologists and has low sensitivity in diagnosing urothelial carcinoma [119]. To this end, a multicentre trial evaluated the diagnostic potential of VisioCyt®, a digital AI-assisted medical device, in voided urine samples obtained from patients with urothelial carcinoma and from negative controls (Fig. 4A) [119, 120]. Notably, VisioCyt® exhibited higher sensitivity compared to conventional cytology carried out by experienced uropathologists [119, 120].

Multiple myeloma (MM) is characterized by the proliferation of malignant plasma cells in the bone marrow [121]. Monoclonal Gammopathy of Undetermined Significance (MGUS) is a premalignant plasma cell dyscrasia associated with elevated levels of IgG immunoglobulin without end-organ damage [121, 122]. To distinguish MM from MGUS at the molecular level, Ruhela and colleagues developed a targeted sequencing panel (295-gene panel for MM) using an AI-driven attention-based model called “Bio-Inspired Graph Network Learning-based Gene-Gene Interaction (BIO-DGI)” [122]. This framework integrated variant profiles such as single nucleotide variants (SNVs), CNVs, structural variants (SVs) and loss-of-function (LOF) variants from WES and WGS datasets of MM and MGUS patients to ultimately identify genes relevant to MM. These combined profiles identified 354 candidate genes which were then filtered to 346 genes after Geo2R validation. After omitting MGUS-specific genes, a list of 282 genes emerged. These genes were categorized into: 70 disease-initiating genes (which were altered in MM and MGUS) and 212 disease-transformative genes (which were specifically altered in MM). Another 13 well-established biomarkers obtained from literature as well as existing MM-related panels were added to formulate the 295-gene panel [122].

A 5-fluorouracil resistance-related signature (5-FRSig) model was constructed to predict the prognosis of colorectal cancer patients and identify the most likely to respond for chemotherapy and immunotherapy [123].

A deep residual learning model predicted microsatellite instability from the H&E stained histology slides of gastrointestinal cancers such as stomach adenocarcinoma and colorectal cancer [124]. Consistently, a DNN identified different tissue types including stromal tissue in the H&E-stained histological images of colorectal cancer which ultimately improved the prediction of the survival of colorectal cancer patients [125]. HIBRID: a DL-based analysis of H&E stained whole slide images combined with circulating tumor DNA (which detects molecular residual disease) enhanced risk stratification in patients with stage II-IV colorectal cancer [126]. Nonetheless, large-scale multicenter prospective validation studies are warranted to support the routine clinical use of this prognostic biomarker.

Although serum prostate-specific antigen (PSA) is commonly used for screening prostate cancer, its compromised sensitivity and specificity lead to unneeded biopsies or false-negative diagnosis [127, 128]. Smelik and colleagues speculated that reliable biomarkers could be spotted after taking into consideration the following (Fig. 4B): i) the distinct grades of malignant transformation within the same tumor. Thus, spots of normal glands, prostatic intraepithelial neoplasia, and five grades of prostate cancer on H&E–stained whole-slide images were annotated and included in the analysis, ii) distinct grades of cellular progression at the molecular level, could be captured and characterized using spatial transcriptomics, and iii) linear trajectory could be constructed using Pseudotime and based on the similarity of the expression profiles of these cells [129]. Notably, 45 genes positively or negatively correlated with the trajectories of Pseudotime. The diagnostic potential of these genes were further assessed in the sera, prostate tissue and urine samples of 2,000 prostate cancer patients and healthy controls. These biomarkers outperformed serum PSA levels in predicting cancer grades. Nonetheless, large-size prospective multicenter studies are urged to validate the sensitivity and specificity of these biomarkers [129].

#### Exploiting AI-driven tools in devising treatment protocols of cancer patients

**Fig. 5:**
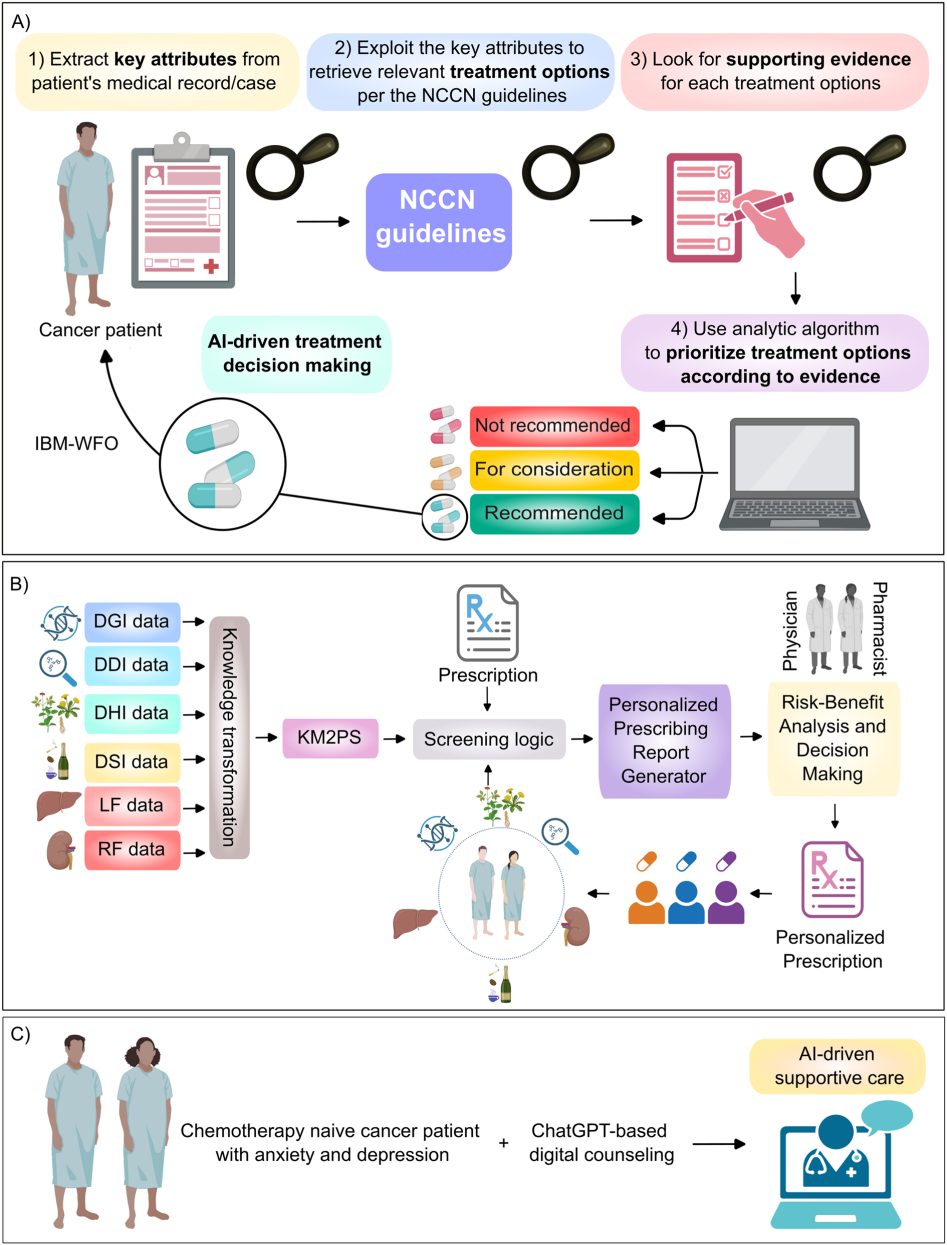
Clinical applications of artificial intelligence (AI)-supported tools in treatment making decisions as well as supportive care of cancer patients. (**A**) Simplified schematic diagram of the workflow of IBM Watson for oncology (WFO), a cognitive computing system which integrated the guidelines of the National Comprehensive Cancer Network (NCCN) for cancer therapy and was trained by the Memorial Sloan Kettering Cancer Center (MSKCC) which exploited natural language processing to find specific attributes in a patient’s record and aimed at guiding the physicians towards evidence-based treatment options ranked into three categories: recommended, for consideration and not recommended [130]. (**B**) Workflow of the knowledge model for potential interaction and potential consideration screening (KM2PS)” [131]. This approach integrated both genetic and non-genetic factors to improve four types of screening before prescribing medication: i) drug-gene interactions (DGI), ii) drug-drug interactions (DDI), iii) drug-herbal interactions (DHI), and iv) drug-social lifestyle interactions (DSI) [131]. This framework also considered the liver function (LF) and renal function (RF). (**C**) Illustrative figure of chemotherapy naïve cancer patients with anxiety and depression who received ChatGPT-based digital counselling to relieve their anxiety and depression. This figure was prepared in part using biorender software

Frameworks powered by AI were used to guide medical oncologists towards rational treatment decisions [130]. Indeed, IBM Watson for Oncology (WFO), a cognitive computing system trained by the Memorial Sloan Kettering Cancer Center (MSKCC), exploited natural language processing to find specific attributes in a patient’s record and ranked evidence-based treatment options into three categories: recommended, for consideration and not recommended (Fig. 5A) [130]. Natural language processing algorithms process and interpret human language [132, 133]. Indeed, WFO has been tested in many countries including the US, China, India and Netherlands. Using retrospective data from 362 cancer patients, Zhou and colleagues examined whether there was a concordance between the treatment decisions proposed by the WFO and that of the multidisciplinary tumor board [130]. Intriguingly, different concordance rates were observed with different cancer types: ovarian cancer (96%), lung cancer and breast cancer ( > 80%), rectal cancer (74%), colon cancer and cervical cancer (64%) and gastric cancer (12%). This discrepancy was speculated to be secondary to different incidence rates and available therapies in the US and China [130].

Since cancer cells depend on multiple driver alterations whose oncogenic effects can be abrogated using combination regimens, Li and colleagues developed a comprehensive resource called “Recurrent Features Leveraged for Combination Therapy (REFLECT)” which integrated ML and cancer informatics algorithms to propose precision combination regimens tailored to target recurrent oncogenic co-alterations across patient cohorts [134].

To avoid potential harm from polypharmacy and foster personalized prescription, Jamrat and colleagues developed the so-called “knowledge model for potential interaction and potential consideration screening (KM2PS)” (Fig. 5B) [131]. To construct KM2PS, knowledge related to potential drug-gene interactions were obtained from distinct tertiary drug information databases including Clinical Pharmacogenetics Implementation Consortium, Pharmacogenomic Knowledge Base, and Table of Pharmacogenomic Biomarkers in Drug Labeling by the US FDA. Knowledge linked to drug-drug interactions, liver functions and renal functions were acquired from Lexicomp, DailyMed, and Drugbank. Drug interactions involving cytochrome P450 were obtained from the Drug Interactions Flockhart Table [131]. Drug-herbal interactions knowledge was obtained from the Medplant database (Mahidol University, Thailand). The input included patient’s prescriptions, medical record, pharmacogenomics data, liver and renal function, the herbal supplements used and social lifestyle (e.g. consumption of caffeine, alcohol and smoking). Thus, this approach integrated both genetic and non-genetic factors to improve four types of screening before prescribing medication: i) drug-drug interactions, ii) drug-gene interactions, iii) drug-herbal interactions, and iv) drug-social lifestyle interactions [131]. This model was proposed to promote risk-benefit analysis and informed clinical decision-making to ensure that the patient receives the optimum personalized treatment (i.e. maximum efficacy and minimum harm) [131].

Ammo and colleagues assessed the decision-making capability of ChatGPT-4o in five simulated cases of sarcoma [135]. Moderate effectiveness of ChatGPT-4o’s recommendations was reported following the evaluation of a panel of an orthopedic surgeon, plastic surgeon, radiation oncologist, radiologist, and pathologist [135].

Within the context of supportive care, a RCT (NCT06854315) assessed the potential effect of a ChatGPT-based digital counselling intervention versus standard clinician-led education group on anxiety and depression in 160 chemotherapy-naïve cancer patients (Fig. 5C) [136]. Intriguingly, the ChatGPT-based counseling significantly mitigated the anxiety and depression in pre-chemotherapy cancer patients compared to the controls [136]. Indeed, further research is required to validate these findings in large-sized multicentre prospective RCTs and refine AI adoption in the supportive care of cancer patients.

Given heightened demand for genetics services and limited availability of certified genetic counselors, women with stage 0-III breast cancer who did not meet the NCCN criteria for genetic testing were randomized to pre-test counselling with a Chatbot or a certified genetic counselor [137]. Of note, there was no significant difference in the median knowledge score and the patient satisfaction score between in-person counselling and automated Chatbot cohorts which would enable better access to healthcare services [137].

**Fig. 6:**
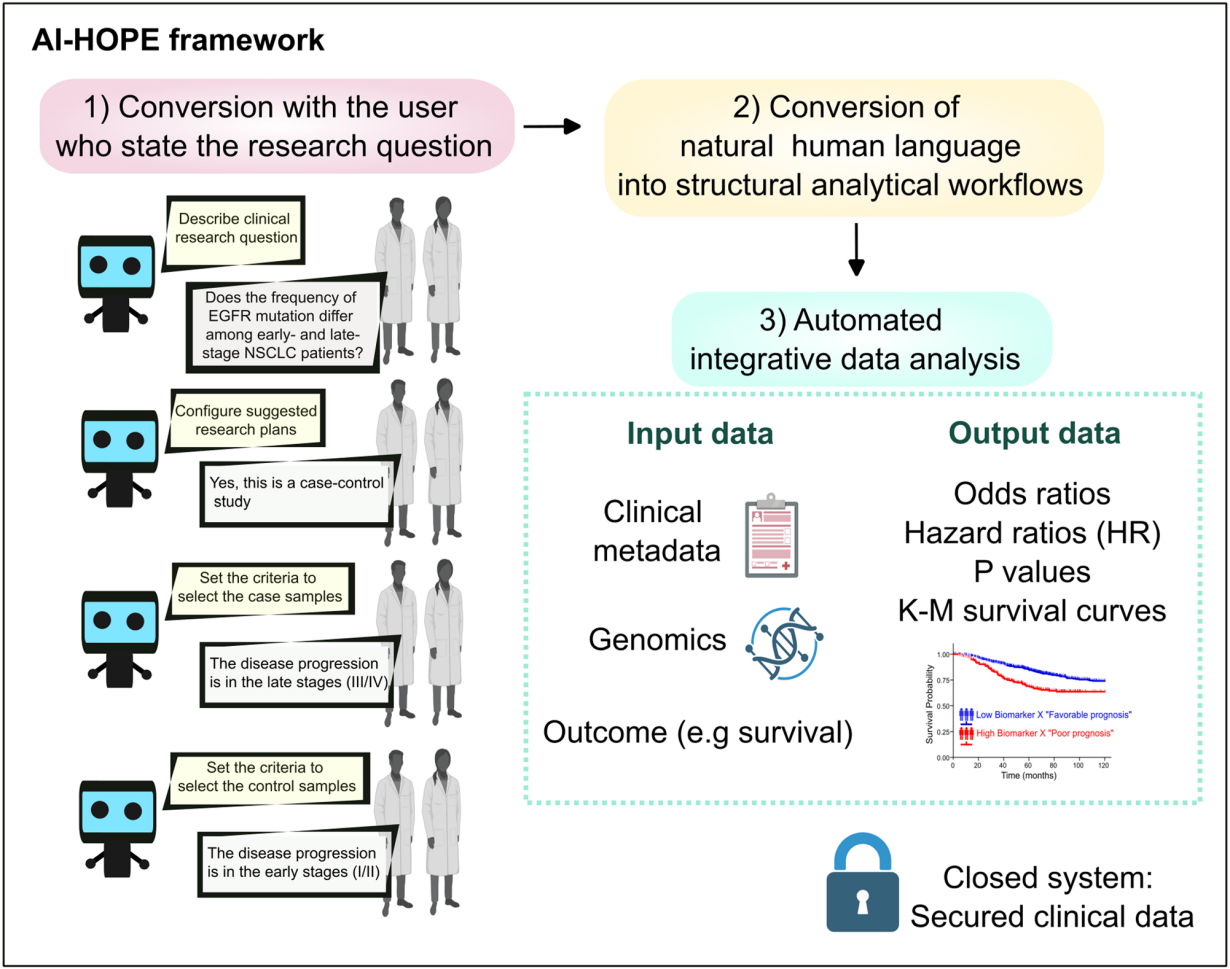
Simplified schematic diagram of the workflow of the artificial intelligence agent for high-optimization and precision medicine (AI-HOPE) [138]. This figure was prepared at least partially using biorender

Many clinical oncologists do not have advanced programming expertise to carry out bioinformatic analyses of large datasets of genomic data, patients’ clinical metadata and treatment outcomes (e.g. survival) [138]. To solve this problem, the Artificial Intelligence Agent for High-Optimization and Precision Medicine (AI-HOPE), an AI-driven conversational agent system was developed (Fig. 6). In this framework, users stated their research question, and AI-HOPE converted natural language into structured analytical workflows. For instance, if the research question was “Do AML patients with FLT3 mutations have different progression-free survival?”, AI-HOPE would initiate a case-control study. On the other hand, if the research question was “Is BRAF mutation in breast cancer linked to brain metastasis?”, AI-HOPE would pursue a data association analysis [138]. AI-HOPE then carried out integrative data analysis of genomics and metadata to generate outputs such as odds ratio, hazard ratios and Kaplan-Meier survival curves. The closed system of AI-HOPE prevented the leakage of clinical data. AI-HOPE pinpointed that TP53 mutations were more prevalent in late-stage (stage III/IV) versus early-stage (stage I/II) colorectal cancer patients [138].

Thus, AI-driven tools have the potential to promote precision cancer medicine via enhancing the diagnosis, screening, biomarker discovery, clinical trial matching, treatment decision and supportive care of cancer patients. Nonetheless, large-size multi-center prospective studies are required to validate the utility of these AI-powered frameworks.

## Limitations of exploiting AI-driven tools in precision oncology and proposed roadmap

Despite the potential of AI-driven tools, there are several challenges which restrain their successful clinical implementation [10, 15, 139]. To accurately and precisely classify or predict tasks, ML and DL algorithms mandate sustainable supply of large datasets [15, 140]. Nonetheless, large datasets are not always readily available and might not be continuously provided to further optimize/train the AI-driven models [15]. This could be in part due to the reluctance of some healthcare institutions to share patients’ data, data privacy issues, compromised data quality and short-term storage of datasets. Modest awareness of the physicians (~25%) about employing AI in medicine was spotted among the challenges of AI in the healthcare industry [141]. Thus, promoting global awareness about the importance of sharing patients’ datasets to train AI-driven models is needed to ultimately improve the clinical outcome of cancer patients. Obtaining patient consent, data encryption and modifying the regulations and rules governing the processing of personal data could also solve data privacy and security issues. The quality of large datasets is usually suboptimal (i.e. they may include missing or inaccurate data). Indeed, accurate assessment of the data labels fed into AI models require multiple subjects to ensure correct data-annotation [140]. Standardization of the electronic health records and interoperability have been proposed to integrate datasets from different healthcare institutes [15]. The relatively short lifespan of stored datasets could be overcome by the cloud computing servers.

Biases (e.g. racial biases) may take place during data collection and hence could compromise the predictive performance of AI-powered models which were trained based on “biased dataset” [15]. To overcome the suboptimal performance of AI-driven algorithms applied to populations different from the training dataset, many researchers diversified their training datasets to be more inclusive and promote health care equity and/or used other technical solutions of generalizability [142]. Low-income and middle-income countries do not have enough technological infrastructure and trained health care providers to access and implement AI-driven technologies [143]. To minimize the AI gap, Khan and colleagues proposed leapfrogging and absorptive strategies for low-income countries with and without strong foundations respectively [144]. AI-leading economies and international organizations such as UNESCO could also help low-income countries catchup with the latest AI technologies through technological cooperation grants, technology transfer, and technical assistance [144].

The so-called “black box” process of AI-driven models also restrained their clinical adoption because it is not always known why these algorithms give a particular prediction, classification or recommendation [15]. The “black box” process of AI-driven model is even more critical when patient-centered AI-driven models goes wrong and the accountability of the physician versus the AI-model developer is questioned. Explainable AI techniques such as Local Interpretable Model-agnostic Explanations (LIME) and Shapley Additive Explanations (SHAP) emerged to clarify how input features contribute to model prediction [145].

The promising results of most AI-assisted tools have been demonstrated in preclinical studies, retrospective studies and in relatively small sized clinical trials [79, 80, 94, 110]. Hence, more large-sized multicentre prospective randomized clinical trials are urged to evaluate the safety and efficacy of AI-assisted tools in precision oncology.

Under-fitting or over-fitting are among the problems of AI-powered models [15]. Under-fitting occurs when the algorithm does not perform well on both the training dataset and validation or unseen data [146]. This could be due to inadequate training or absence of relevant predictive features. Conversely, over-fitting takes place when an algorithm performs well on training data but not on unseen or external data [146]. This happens when the model learns irrelevant relationships between the inputs (e.g. patient variables) and the outcomes or when the model predicts inaccurate future outcomes using unsuitable features (for instance when there are many variables compared to the outcomes) [15]. Validation of AI-driven models could be carried out using external datasets and large-size prospective trials involving national and international organizations [15, 140]. Thus, AI-driven training algorithms should be validated in different sites to enhance their generalizability.

AI-driven platforms are not formulated to replace clinicians but rather to spare their efforts and time to do more sophisticated human intellectual tasks which cannot be carried out by machines. Thus, developers of AI-driven algorithms should take into consideration that these models would be ultimately integrated into the workflow of medical oncologists; the majority of which may have limited programming skills [15]. Thus, the user interface of these tools should be user friendly, time-saving and easily interpretable.

Implementing AI-driven tools in clinical practice mandates systematic coordination between several stakeholders including governments, public sector agencies, AI-developing industries, researchers, and implementers. The World Health Organization (WHO) has published a comprehensive guidance on the ethics and governance of AI for health [147]. This guidance presented six principles: i) protecting autonomy; ii) advocating human well-being and safety; iii) promoting transparency, explainability and intelligibility; iv) promoting responsibility and accountability; (v) fostering inclusiveness and equity; and vi) ensuring the responsiveness and sustainability of AI [147].

Approximately 1000 AI-powered medical devices have been authorized by the US FDA which continue to receive hundreds of regulatory submissions for small molecules which were discovered and developed using AI-powered tools [148]. The US FDA released a guidance entitled “Considerations for the Use of Artificial Intelligence to Support Regulatory Decision-Making for Drug and Biological Products Guidance for Industry and Other Interested Parties” [149]. In this draft, the FDA presented a risk-based credibility assessment framework that could be exploited for assessing the credibility of AI-driven models. This guidance reviewed the deployment of AI-powered algorithms in the drug product life cycle to support regulatory decision-making linked to the safety or effectiveness of drugs [149]. This includes using AI-powered models in: i) minimizing the number of in vivo pharmacodynamic, pharmacokinetic and toxicological studies, ii) predicting the clinical pharmacokinetics and driving exposure-response analyses, iii) multimodal data integration from distinct sources, iv) defining and assessing post-marketing adverse drug experience information, and v) identifying optimum manufacturing conditions. It is worth noting that the US FDA underscored the importance of maintaining the credibility of the AI-driven models because their performance can vary over time [148]. Kore and colleagues shed light on the significance of monitoring the clinically deployed AI-driven models for data drift [142]. The term “data drift” indicates systematic alteration in the distribution of the input features (e.g. changes in the demographics of a population following immigration) which could compromise the performance of the deployed AI-driven model [142]. Data drifts were affected by the sample size and patients features [142]. Monitoring deployed AI models for data drifts could help act before the safety of the patient is compromised and decide whether the existing model should be re-assessed, re-trained, suspended or replaced [142].

The UK National Health Service (NHS) also developed the ‘Code of Conduct for Data-Driven Health and Care Technologies’ in which they provided principles linked to data ethics framework, usability and accessibility, technical assurance, clinical safety, data protection and transperancy, cybersecurity, regulatory requirements, interoperability and open standards [150].

To sum, our expectations regarding the potential of AI-driven tools in precision oncology should be realistic. AI is dynamic in nature with more precise and deeper models being developed overtime. Thus, adoption of AI-powered algorithms in clinical practice merits more time and solid evidence proving their safety and efficacy.

## Conclusions and future directions

“One-size-fits-all” therapies failed to induce complete remission in a significant proportion of the treated cancer patients. Hence, precision and personalized medicine is foreseen to revolutionize cancer therapy. Precision oncology necessitates sophisticated analyses. AI-driven tools have the potential to fill the gap by enabling integrative analysis of massive data including that of multi-omics data, clinical metadata, radiomics features and chemical features of anticancer drugs. The multi-spectrum potential of AI-driven tools in optimizing almost all stages of the preclinical cancer research starting from discovery of druggable cancer targets, target pocket prediction, structure-based and ligand-based virtual screening, hit discovery, lead optimization, generation of novel small molecules as well as scaffold hopping and prediction of cancer drug response. There are many small molecules which demonstrated potent anticancer activity yet still have unknown direct target(s) [104, 151, 152]. The process of identification of the precise target(s) is sophisticated, time-consuming and costly [153]. By multimodal analysis of drug response data, drug modeling data and cancer cell modeling data, AI-driven models could help predict the potential target(s) of the small molecules. Within the clinical oncology context, AI-based frameworks integrating patient’s medical record, image features and multi-omics data promoted the identification of predictive, prognostic, susceptibility and diagnostic biomarkers, supported clinical trial matching, guided informed treatment decision making and provided supportive care for cancer patients. However, large-size multicenter prospective RCTs are needed to confirm the potential added value of AI-powered tools in managing diverse types of cancer, especially rare cancers and across all ethnicities. Other limitations include data security and privacy, potential bias in the data used, model accuracy, unfair accessibility to precision and personalized medicine, need for advanced programming skills, difficulty of interpreting AI-driven data, and need for clinical real-world validation. Indeed, consensus regulatory standards and ethical guidelines for using AI-driven models are warranted to prevent data leakage and ensure responsible and effective use of AI-powered tools to benefit all cancer patients rather than the privileged subset only. Notably, more “user-friendly” and “publicly available” AI-assisted frameworks covering their different applications are needed to reduce the technical and financial barriers and ultimately help scientists, medicinal chemists, pharmacologists and medical oncologists to unleash the full potential of AI-assisted algorithms in preclinical cancer research and in clinical practice. This would be of paramount importance especially for research institutions and healthcare systems in low-income and middle-income countries with limited resources. Thus, we are currently at the tip of the iceberg of “AI-powered precision cancer medicine” and more systematic efforts are needed to fully exploit its capabilities.

## Electronic supplementary material

Below is the link to the electronic supplementary material.

Supplementary Material 1

## Data Availability

No datasets were generated or analysed during the current study.

## Abbreviations

AAC: Area above the dose response curve
ABL1: Abelson murine leukemia viral oncogene homolog 1
ADC: Antibody-drug conjugate
AI: Artificial intelligence
AI-HOPE: Artificial intelligence agent for high-optimization and precision medicine
AML: Acute myeloid leukemia
ANN: Artificial neural network
AUC: Area under the curve
AURKA: Aurora Kinase A
BCR: Breakpoint cluster region
CACNA2D1: Voltage-dependent calcium channel subunit alpha-2/delta-1
CAR: Chimeric antigen receptor
CCLE: Cancer Cell Line Encyclopedia
CDRScan: Cancer Drug Response profile scan
CML: Chronic myeloid leukemia
CNN: Convolutional neural network
CPIC: Clinical pharmacogenetics implementation consortium
CRISPR: Clustered Regularly Interspaced Short Palindromic Repeats
CTLA4: Cytotoxic T-lymphocyte-associated protein 4
CTRP: Cancer Therapeutics Response Portal
DL: Deep learning
DNMT: DNA methyl transferase
DNN: Deep neural network
DIPK: Deep neural network Integrating Prior Knowledge
EBV: Epstein-Barr virus
EGFR: Epidermal growth factor receptor
FDA: Food and Drug Administration
FLT3: FMS-like tyrosine kinase 3
G6PD: Glucose-6-phosphate dehydrogenase
HER2: Human epidermal growth factor receptor 2
ICI: Immune checkpoint inhibitor
KM2PS: Knowledge model for potential interaction and potential consideration screening
LOF: Loss of function
MAPK: Mitogen activated protein kinase
MGUS: Monoclonal Gammopathy of Undetermined Significance
ML: Machine learning
MM: Multiple myeloma
MRI: Magnetic resonance imaging
NGS: Next generation sequencing
NSCLC: Non-small cell lung cancer
NUDT15: Nudix hydrolase 15
PD1: Programmed cell death protein 1
PDL1: Programmed cell death ligand 1
PSA: Prostate-specific antigen
RCT: Randomized controlled trial
RT-DMF: Residual threshold deep matrix factorization
SV: Structural variants
TCGA: The Cancer Genome Atlas
TKI: Tyrosine kinase inhibitor
TPMT: Thiopurine methyl transferase
VEGF: Vascular endothelial growth factor
